# The molecular mechanisms that underlie fragile X-associated premature ovarian insufficiency: is it RNA or protein based?

**DOI:** 10.1093/molehr/gaaa057

**Published:** 2020-08-10

**Authors:** Roseanne Rosario, Richard Anderson

**Affiliations:** MRC Centre for Reproductive Health, Queens Medical Research Institute, University of Edinburgh, Edinburgh, UK

**Keywords:** FXPOI, molecular mechanisms, RNA gain-of-function, RAN translation

## Abstract

The *FMR1* gene contains a polymorphic CGG trinucleotide sequence within its 5′ untranslated region. More than 200 CGG repeats (termed a full mutation) underlie the severe neurodevelopmental condition fragile X syndrome, while repeat lengths that range between 55 and 200 (termed a premutation) result in the conditions fragile X-associated tremor/ataxia syndrome and fragile X-associated premature ovarian insufficiency (FXPOI). Premutations in *FMR1* are the most common monogenic cause of premature ovarian insufficiency and are routinely tested for clinically; however, the mechanisms that contribute to the pathology are still largely unclear. As studies in this field move towards unravelling the molecular mechanisms involved in FXPOI aetiology, we review the evidence surrounding the two main theories which describe an RNA toxic gain-of-function mechanism, resulting in the loss of function of RNA-binding proteins, or a protein-based mechanism, where repeat-associated non-AUG translation leads to the formation of an abnormal polyglycine containing protein, called FMRpolyG.

## Introduction

**Figure 1.**
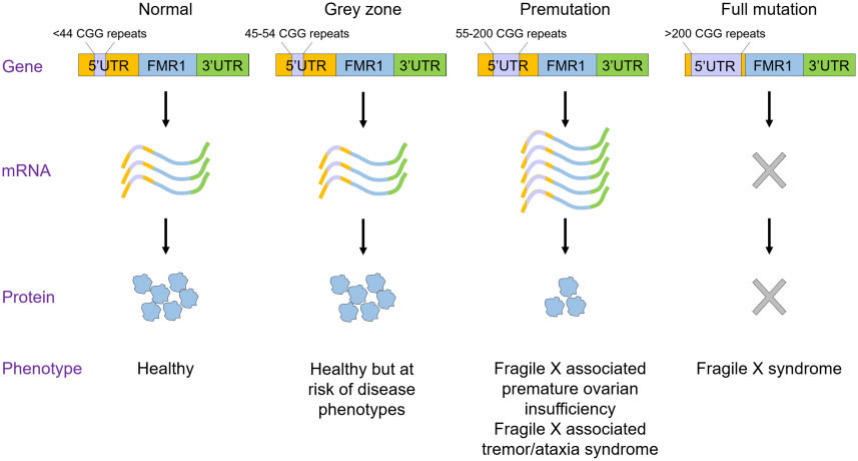
**CGG repeat length is polymorphic.** Normal individuals have fewer than 44 CGG repeats (mean 29) and CGG repeat lengths between 45 and 54 are termed grey zone, as they are at risk of developing disease phenotypes due to CGG repeat instability. Individuals with a premutation have between 55 and 200 CGG repeats, elevated *FMR1* mRNA levels and reduced FMRP protein levels, and the premutation causes the conditions fragile X-associated premature ovarian insufficiency and fragile X-associated tremor/ataxia syndrome. Individuals with more than 200 CGG repeats have a full mutation, where the *FMR1* gene is transcriptionally silenced, leading to an absence of *FMR1* mRNA and FMRP protein. The full mutation causes fragile X syndrome. Figure adapted from Hagerman and Hagerman (2002).

Fragile X-associated premature ovarian insufficiency (FXPOI) is among a family of disorders caused by the expansion of a CGG trinucleotide repeat sequence located in the 5′ untranslated region (UTR) of the *FMR1* gene on the X chromosome. This repeat sequence is polymorphic in length, with categorization into four different size ranges (Fig. 1). Healthy individuals have a normal repeat length with fewer than 44 CGG repeats, while having between 45 and 54 repeats is classified as intermediate, or grey zone. Although this sequence length is not directly associated with disease phenotypes, some level of CGG repeat instability has been reported, which results in variable repeat expansion during mother-offspring transmission (Nolin *et al.*, 1996). The range of 55–200 CGG repeats is considered a premutation, and more than 200 CGG repeats are categorized as a full mutation. Full mutations underlie the severe neurodevelopmental condition fragile X syndrome (Kronquist *et al.*, 2008), which is the most common cause of inherited intellectual disability and autism in males, with patients suffering from a wide range of clinical, cognitive and behavioural dysfunctions. While premutation carriers were originally thought to be clinically unaffected, it was noted that some females in families with a fragile X male experienced a premature menopause, now characterized as FXPOI (Cronister *et al.*, 1991; Conway *et al.*, 1998). Premutations also result in the more recent described fragile X-associated tremor/ataxia syndrome (FXTAS), a multisystem neurological disorder with tremor and ataxia as its principal features, which was initially recognized in ageing carriers but with clinical features potentially also present in children (Hagerman *et al.*, 2001). Much of the research into the molecular mechanisms underlying the pathology of *FMR1*-associated conditions has focused on the neurological aspects: the aim of this review is to critically appraise putative mechanisms underlying the pathogenesis of FXPOI, specifically the evidence investigating the RNA gain-of-function hypothesis and the contribution of repeat-associated non-AUG (RAN) translation, drawing on parallels in FXTAS and advances made in that disease.

## Fragile X-associated premature ovarian insufficiency

Premature ovarian insufficiency (POI) is defined as the loss of ovarian hormonal function with evidence of follicle depletion before the age of 40 years (Welt, 2008). It thus has a direct impact on fertility and secondary consequences arising from oestrogen deficiency, such as osteoporosis and bone fractures (Gallagher, 2007), impaired endothelial function (Kalantaridou *et al.*, 2004), earlier onset of coronary heart disease and increased cardiovascular mortality (Mondul *et al.*, 2005) (see Webber *et al.*, 2016 for a comprehensive review). In addition, women who have a premature menopause are reported to suffer from more anxiety, depression and psychological distress than women with normal ovarian function (van der Stege *et al.*, 2008; Hunter *et al.*, 2010). Identifiable causes of POI include iatrogenic causes, often secondary to the treatment of cancer, and autoimmune causes. Genetic causes remain largely enigmatic, despite the identification of a substantial number of genes necessary for normal ovarian function from murine knock-out studies (Matzuk, 2000). A clear familial pattern is found in some cases even without syndromic conditions such as blepharophimosis/ptosis/epicanthus inversus syndrome, which is associated with mutations in the granulosa cell-specific gene *FOXL2* (Crisponi *et al.*, 2001). The X chromosome carries many genes that govern follicular maturation and overall ovarian function, and numerical and structural changes in this chromosome, as in Turner syndrome or triple X syndrome, are associated with POI (reviewed in Fortuño and Labarta, 2014). Abnormalities in the X-linked *FMR1* gene were the first causative genetic abnormality identified and are the only monogenic cause currently tested for in routine clinical practice, as POI occurs in about 20% of *FMR1* premutation carriers compared with approximately 1% of all women in the general population (Sherman *et al.*, 2014). CGG repeat length shows a strong non-linear association with penetrance for POI (Sullivan *et al.*, 2005; Ennis *et al.*, 2006; Allen *et al.*, 2007; Tejada *et al.*, 2008). In the general population, 29 CGG repeats are the most frequent length observed (Fu *et al.*, 1991). Women carrying midrange repeats (approximately 70–90 repeats) have the highest risk for POI, while carriers of smaller and larger premutation repeat lengths have an increased risk of POI compared to the general population, but not to the same extent as mid-range carriers. It remains contentious whether normal or grey zone CGG repeat lengths are correlated with ovarian reserve parameters, age at natural menopause and IVF outcome (Gleicher *et al.*, 2009; Gleicher *et al.*, 2011; Voorhuis *et al.*, 2013; Gustin *et al.*, 2015; Banks *et al.*, 2016; Pastore *et al.*, 2019). In addition, X chromosome inactivation (Sullivan *et al.*, 2005; Tejada *et al.*, 2008; Spath *et al.*, 2010), background genes (Hunter *et al.*, 2008; Spath *et al.*, 2011) and smoking (Spath *et al.*, 2011) have all been investigated to explain the incomplete penetrance of POI among premutation carriers.

For many women, the most immediate and significant consequences of POI are amenorrhoea and an inability to conceive, however, some women present with intermittent ovarian function and conceive naturally (Rebar *et al.*, 1982; Fraison *et al.*, 2019); thus women with FXPOI may have a child with fragile X syndrome due to CGG repeat instability (Nelson *et al.*, 2005). Female relatives may also be asymptomatic carriers of expanded repeat number, with consequences for themselves and future potential offspring. Although normal-length repeats are usually transmitted in a stable manner, the risk of expansion increases with the length of the repeat, with grey zone-length repeats expanding to full mutation within several generations, and premutations having the highest risk, with expansion to full mutation in one generation (Nolin *et al.*, 2003; Fernandez-Carvajal *et al.*, 2009). Here, the sex of the transmitting parent is important; while the risk of expansion of grey zone alleles is higher for fathers than mothers, the expansion of premutation alleles is limited to mothers only (Sullivan *et al.*, 2002). Furthermore, the number and position of AGG nucleotides, which usually interrupt the CGG repeat tract (CGG repeats are not ‘pure’) have also been explored. In their absence, the length of pure CGG is increased and is more prone to replication slippage and subsequent expansion (Yrigollen *et al.*, 2012; Nolin *et al.*, 2015). AGG interruptions have also been directly correlated with parameters of the ovarian reserve, with patients having longer uninterrupted CGG repeats post-AGG interruptions having the lowest anti-Müllerian hormone levels and antral follicle counts (Lekovich *et al.*, 2018).

## Clues to the aetiology of FXPOI: physiological and molecular mechanisms

The physiological mechanisms that underlie compromised follicular function preceding the full development of FXPOI are unclear, but it is proposed these insults could occur at various stages of follicular development. These include (i) inadequate formation of the ovarian reserve during foetal life and (ii) alterations in follicle recruitment and maturation that cause abnormally extensive or fast depletion of the ovarian reserve during post-natal (neonatal, childhood and adult) life. While information from women is scarce, the development and use of model systems has gone some way towards addressing this, with data available from three published knock-in mouse strains, harbouring 130 (here, ‘130R’) (Hoffman *et al.*, 2012), 90 (here, ‘90R’) (Lu *et al.*, 2012) and 100-199 (here, ‘Dutch’) (Bontekoe *et al.*, 2001) CGG repeats in the *FMR1* 5′UTR, respectively (see Berman *et al.*, 2014; Sherman *et al.*, 2014 for comprehensive reviews on these models). There is mostly consensus in the reproductive physiology between the 130R and 90R strains, which show that the premutation allele does not hinder the establishment of the primordial follicle pool, but rather affects ovarian follicle growth and survival, with a parallel decrease in litter size (Pastore and Johnson, 2014). Additionally, the growth of ovarian follicles is slower due to an elevation in the follicular apoptotic index (Uslu *et al.*, 2017), with a decrease in the number of cumulus granulosa cells associated with mature and ovulatory follicles. Both 130R and 90R models are associated with increased levels of follicular atresia and this follicular decline appears to affect each follicle stage equally (Hoffman *et al.*, 2012; Lu *et al.*, 2012), in contrast to what is seen in many mutant mice with impaired fertility where specific follicle stages are often affected. This compromised follicle function was found to correlate with reduced mitochondrial DNA copy number, total mitochondrial mass and altered expression of genes that control mitochondrial function (Conca Dioguardi *et al.*, 2016), and while mitochondrial abnormalities have been detected in premutation carriers (Alvarez-Mora *et al.*, 2017), causal links between the premutation, mitochondria and follicle function remain to be elucidated. While granulosa cell abnormalities may be one source of reduced follicle viability, oocytes from premutation mice have aberrant localization of the fragile X mental retardation protein (FMRP), with much higher FMRP levels in the nucleus than in the cytoplasm compared with wildtype mice (Hoffman *et al.*, 2012). Given that FMRP has been suggested to have a function in the nucleus (Bagni and Greenough, 2005), increased expression within the nucleus may be deleterious if it causes dysregulation of this function. Furthermore, oocytes of premutation mice also have increased ubiquitin levels, which may indicate a reduced efficiency of the proteasome system for protein degradation or an increased accumulation of abnormal proteins targeted for degradation by ubiquitin (Hoffman *et al.*, 2012). Interestingly, an ovarian phenotype has also been observed in the full mutation knockout mouse model, with *FMR1* knockout mice having an increased number of follicles and larger ovaries than wildtype mice, with corpora lutea indicating ovulation (Ascano *et al.*, 2012). Here, the authors suggest that loss of FMRP may lead to precocious follicular development, although while this model may have the potential to recapitulate ovarian insufficiency through follicle over-activation; whether this mechanism is associated with FXPOI is an unanswered question. However, women who carry the full mutation do not appear to be at increased risk of POI; whether this is because they are heterozygous for the loss of FMRP is unknown (Sherman *et al.*, 2014).

**Figure 2.**
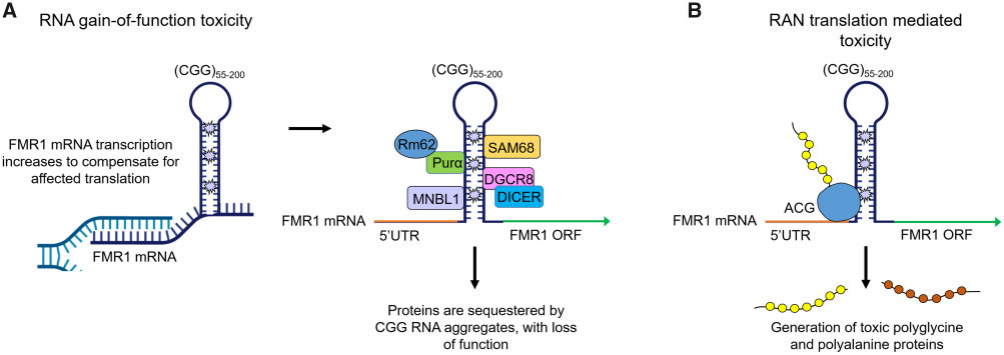
**RNA gain-of-function toxicity and RAN translation-mediated toxicity.** (**A**) RNA gain-of-function toxicity. *FMR1* transcription increases to compensate for affected translation. Subsequently, premutation CGG repeat lengths form intranuclear aggregates that can sequester RNA binding proteins, inhibiting them from carrying out their normal roles, leading to cell dysfunction. (**B**) Repeat-associated non-AUG (RAN) translation-mediated toxicity. Translation of *FMR1* mRNA is initiated from a near cognate ACG start codon, resulting in the production of polyglycine and/or polyalanine-containing proteins that interfere with normal cell function or might be directly toxic. Figure adapted from Berman *et al.* (2014).

Indeed, and perhaps paradoxically, clues to the aetiology of FXPOI at the molecular level may lie in the fact that women carrying full mutations do not develop POI. In *FMR1* full mutations, the expanded CGG repeat is recognized as a CpG island resulting in methylation, chromatin condensation and transcriptional silencing of *FMR1* and thus a loss of FMRP (Warren, 2007). Two novel regions named FREE1 and FREE2 have also been identified as having a role in the epigenetic regulation of *FMR1* (Godler et al., 2010). Largely located within intron 1, FREE1 and FREE2 are hypermethylated in fragile X individuals but unmethylated in carriers of smaller expansions. FMRP is a ribosome-associated RNA-binding protein that interacts with the coding region of transcripts encoding pre- and post-synaptic proteins implicated in autism spectrum disorders. FMRP reversibly stalls ribosomes on its target mRNAs, and loss of this translational brake on a subset of synaptic proteins contributes to fragile X syndrome (Darnell *et al.*, 2011). Indeed, some authors (Gleicher *et al.*, 2009; Schuettler *et al.*, 2011) speculate that reductions in FMRP expression in granulosa cells as a result of the premutation might directly impact the ovarian reserve through increased follicle activation, as FMRP may translationally regulate paracrine signals and signalling pathways that are important for the regulation of the initiation of follicle growth. In particular, data from the ‘90R’ premutation mouse showed reduced expression of phosphorylated mTOR (Lu *et al.*, 2012), while the *Fmr1* knockout model had elevated mTOR levels (Ascano *et al.*, 2012). Dysregulation of mTOR signalling can result in ovarian dysfunction, with inhibition of mTOR causing reduced granulosa cell proliferation (Yu *et al.*, 2011), which is a notable phenotype in the premutation mouse models. Furthermore, experiments in human granulosa cell line COV434 allude to potential interactions between AKT/mTOR signalling and *FMR1* (Rehnitz *et al.*, 2017)*.* While significant efforts have been made to uncover and validate the mRNA targets of FMRP in the brain (Darnell *et al.*, 2005; Darnell *et al.*, 2011; Bagni *et al.*, 2012; Suhl *et al.*, 2014; Maurin *et al.*, 2018), there has been little progress regarding the identification of the direct translational targets of FMRP within the ovary. However, in premutation and grey zone carriers, *FMR1* is transcriptionally active and mRNA levels are actually elevated (Tassone *et al.*, 2007), with reports describing a correlation between increasing repeat length, increasing *FMR1* transcript levels, and decreasing FMRP levels. Thus a key hypothesis is that *FMR1* mRNA gain-of-function toxicity may underlie FXPOI, as is thought to be the case in the other premutation associated disorder, FXTAS (Hagerman and Hagerman, 2002) (Fig. 2A). An alternative hypothesis has also been proposed, based on the observation that repeat sequences can increase the frequency at which translation initiates at non-AUG start codons, a process known as RAN translation. These false start sites result in polyglycine and/or polyalanine-containing proteins that have been shown to be neurotoxic and have been detected in the brains of FXTAS patients (Todd *et al.*, 2013) (Fig. 2B).

## RNA gain-of-function toxicity: does it underlie FXPOI?

Several pathologies, such as Huntington’s disease and myotonic dystrophy, are caused by trinucleotide expansions, and these expansions can result in either a loss or gain-of-function of the translated protein. Fragile X syndrome is an example of where the expansion has a loss-of-function mechanism at the protein level, as the trinucleotide repeat results in lower or absent levels of the gene product, FMRP (Warren, 2007). When there is a gain-of-function, mRNA or protein are thought to acquire a new cellular function depending on the extent and localization of the expansion. In FXTAS, ubiquitin-positive intranuclear inclusions comprised of mRNA and protein are observed in the brain (Galloway and Nelson, 2009), and *FMR1* mRNA can be found within these inclusions (Tassone *et al.*, 2004). This, together with the elevated expression of expanded *FMR1* mRNA in premutation carriers (Tassone *et al.*, 2000a), gave rise to the hypothesis that a toxic mRNA gain-of-function effect might be responsible for the development of FXTAS (Tassone *et al.*, 2000a,b; Hagerman *et al.*, 2001; Greco *et al.*, 2002). This concept originated from the pathogenesis of myotonic dystrophy, where a CUG triplet expansion in the promoter of the DMPK gene leads to the formation of intranuclear inclusions. These inclusions sequester the RNA-binding protein MBNL (muscleblind-like), leading to dysregulated splicing of MBNL targets and thus clinical symptoms through a deficit of affected proteins (Fardaei *et al.*, 2001; Kanadia *et al.*, 2003). If this mechanism does contribute to FXTAS pathogenesis, its involvement in FXPOI may also be plausible. 

According to this hypothesized model for *FMR1* premutations, expanded CGG-containing transcripts are exported from the nucleus in messenger ribonucleoprotein complexes, however, these transcripts do not join the 40S ribosomal subunit and consequently translation is affected, reducing FMRP levels. To compensate, *FMR1* transcription is augmented and consequently, there is an increase in *FMR1* mRNA levels (Oostra and Willemsen, 2003). Cells attempt to eliminate surplus *FMR1* transcripts via the ubiquitin-proteasome degradation pathway (Barasoain *et al.*, 2016) however when this is not achieved, intranuclear inclusions containing CGG-repeat mRNA are formed. Simultaneously, this CGG-repeat mRNA is known to adopt secondary structures, such as intramolecular hairpins (Zumwalt *et al.*, 2007), thus RNA-binding proteins could bind to these non-canonical RNA structures, forming RNA-protein aggregates in cells (Fig. 2A). Sequestration of these RNA-binding proteins inhibits them from their normal functions, compromising cell integrity and potentially causing cell death. It is important to note that in this model, toxicity arises as a result of the expanded CGG repeat itself, and not of overexpression of *FMR1* protein product, as overexpression of *FMR1* mRNA without a CGG repeat expansion does not trigger neuronal death or produce behavioural deficits (Fernández *et al.*, 2012). While it was initially suggested that the toxic effect of the elevated levels of *FMR1* mRNA could lead to a diminished ovarian reserve before birth (Conway *et al.*, 1995), and indeed *FMR1* is expressed in germ cells in the human foetal ovary (Rosario *et al.*, 2016), it has since been proposed that ovarian dysfunction associated with premutation alleles could be considered a late age-of-onset disease, with mRNA toxic effects causing increased atresia of follicles throughout the lifetime (Sullivan *et al.*, 2005). Indeed, evidence from premutation mouse models is in line with this idea. Comparably, women with type 1 myotonic dystrophy have evidence of a diminished ovarian reserve (Srebnik *et al.*, 2014), thus the accumulation of RNA-protein complexes thought to underlie that disease could perhaps also occur in the ovary.

Alternative mechanisms proposed to describe the toxicity of premutation RNA have suggested that expanded-repeat RNA may separately elicit a stress response, evidence of which has been observed both in brain tissue of premutation carriers (Chen *et al.*, 2010) and a *Drosophila* model of repeat RNA toxicity, where the innate immune response is activated (Samaraweera *et al.*, 2013). It is thought that RNA secondary structures may arise in mid-range CGG repeat carriers with reduced AGG interruptions, which could cause cell dysfunction within the ovaries resulting in the diminished ovarian reserve observed in these specific carriers (Lekovich *et al.*, 2018). Indeed, hairpin secondary structures formed from repeat CGG RNA can act as a substrate for the enzyme Dicer (Handa *et al.*, 2003), which is responsible for generating small RNAs that act via the RNA interference pathway post-transcriptionally to reduce the expression of complement target genes. While small RNAs that are toxic to cultured neuronal cells have been generated from expanded CAG repeats (Samaraweera *et al.*, 2013), it remains to be seen whether repeat-CGG RNA can be toxic in the same way. Similarly, increased amounts of *FMR1* mRNA in granulosa cells could also lead to a rise in R-loop formation, a secondary DNA-RNA hybrid structure formed by the CGG repeats. The presence of R-loops could lead to increased DNA damage within the cell, because in this structure DNA is more unstable and susceptible to mutagenesis and genomic instability (Aguilera, 2002; Gan *et al.*, 2011; Man *et al.*, 2017). While these structures have been observed in cultured human dermal fibroblasts containing the premutation (Loomis *et al.*, 2014), no R-loops have been detected in human granulosa cells.

Nevertheless, in support of the *FMR1* premutation model and RNA gain-of-function hypothesis, increased expression of *FMR1* mRNA has been observed in the ovaries of all premutation mouse models, with expression being localized to both granulosa cells and oocytes (Sherman *et al.*, 2014). Furthermore, although FMRP levels differ among the premutation mouse models, likely due to their genetic construction, both the 130R and 90R mice have reduced FMRP expression (Sherman *et al.*, 2014). In humans, FMRP is expressed in oocytes of foetal ovaries and in granulosa cells of mature follicles (Rife *et al.*, 2004; Rosario *et al.*, 2016), and increased *FMR1* transcript levels have been reported in granulosa cells of premutation carriers (Elizur *et al.*, 2014). In that work, the authors also describe a significant negative linear correlation between granulosa cell *FMR1* expression and the number of oocytes retrieved after ovarian stimulation for preimplantation genetic diagnosis, with the most obvious effects observed in women carrying mid-range (80–120) repeat lengths. While these data are only correlative, these findings suggest that *FMR1* mRNA accumulation in granulosa cells may be an important cause of follicle dysfunction and loss. A preliminary report of *in vitro*-based studies further demonstrated that transfection of the human granulosa cell tumour line COV434 with plasmid DNA expressing 88 CGG repeats decreased cell viability after 48 h (Hubayter *et al.*, 2009), also supporting a proposed toxic RNA gain-of-function mechanism.

Identification of the proteins that bind and are sequestered by CGG RNA within intranuclear inclusions in the brain has been an important step towards understanding the contribution of RNA gain-of-function toxicity to the pathogenesis of FXTAS. Several proteins have been identified that bind to CGG repeats in FXTAS-affected cells, unlike other disorders associated with unstable repeat expansions, which usually have a principle protein deposit (Loesch and Hagerman, 2012). RNA binding proteins that have been shown to interact directly with CGG-repeat RNA include hnRNP A2/B1, a protein involved in pre-mRNA processing (Jin *et al.*, 2007; Sellier *et al.*, 2010), Purα, which is associated with transcription regulation in neuronal development (Sellier *et al.*, 2010), and the RNA splicing protein MBNL1, a protein also implicated in the pathogenesis of myotonic dystrophy (Loesch and Hagerman, 2012). The RNA binding protein DGCR8 has also been demonstrated to bind to expanded CGG repeats, resulting in its partial sequestration with its protein partner DROSHA, a key enzyme that executes the initiation step of miRNA processing in the nucleus (Sellier *et al.*, 2013). Consequently, miRNA processing was reduced, as evidenced by decreased levels of mature miRNAs in neuronal cells expressing CGG repeats and brain tissue from FXTAS patients, suggesting that the dysregulation of miRNAs may be involved in premutation-related toxicity. Overexpression of hnRNP (Sofola *et al.*, 2007), Purα (Jin *et al.*, 2007) and DROSHA (Sellier *et al.*, 2013) can rescue neurodegeneration in a fly model of FXTAS, however, whether they can rescue the mammalian phenotype remains to be seen. Importantly, CGG RNA aggregates have been shown to be dynamic structures that expand in size and number over time (Sellier *et al.*, 2010), thus while some proteins are initially sequestered by RNA aggregates in a specific manner, these proteins in turn are able to recruit additional proteins. These include CUGBP1 in the case of hnRNP A2/B1 (Kuyumcu-Martinez *et al.*, 2007) and the RNA helicase Rm62 in the case of Purα (Qurashi *et al.*, 2011), ultimately causing widespread cellular dysfunction. With regards to relevance for FXPOI, the identification of SAM68 in CGG RNA aggregates is particularly meaningful. Although the authors used a cell line model of the premutation to demonstrate sequestration of SAM68, and consequent loss of its normal alternate splicing function (Sellier *et al.*, 2010), SAM68 has been suggested to regulate the mRNA splicing of FSH and LH receptors (Bianchi *et al.*, 2010). Sam68^−/−^ female mice are severely subfertile and morphological analyses of the ovary indicated a significant reduction in the number of secondary and pre-antral follicles. Crosslinking/immunoprecipitation experiments showed that Sam68 directly binds FSH and LH receptor mRNAs, which were down-regulated in ovaries of adult knockout mice. Indeed, altered splicing of these proteins in FXPOI-affected granulosa cells could lead to FSH and LH resistance at the receptor level, preventing follicle maturation (albeit that primordial follicle loss rather than gonadotrophin resistance is the ovarian phenotype in FXPOI). However, although the proteins discussed here are predicted to be also relevant in the pathogenesis of FXPOI, there has been no validation as yet of these proteins in ovary-specific premutation models. Furthermore, while a degree of overlap is expected given the parallels with FXTAS, it is probable that some deregulated proteins will be specific to granulosa cells, thus identification of these ovarian targets will be essential as they are likely to be key in understanding the RNA gain-of-function mechanism that may potentially contribute to FXPOI.

Finally, the discovery of long non-coding (lnc) RNAs originating from the *FMR1* locus also supports RNA toxic gain-of-function as one of the possible pathophysiologic mechanisms underlying FXPOI. Using genomic approaches (Khalil *et al.*, 2008), and more recently, ‘Deep-RACE’ methodology (Pastori *et al.*, 2014), the *FMR1* gene locus was interrogated for the occurrence of novel lncRNAs, leading to the discovery of *FMR4*, *FMR5* and *FMR6*. While *FMR5* appeared to show no differential expression between unaffected, premutation and full mutation carriers (Pastori *et al.*, 2014), *FMR4* was silenced in full mutation carriers and up-regulated in premutation carriers, similar to *FMR1* (Khalil *et al.*, 2008)*.* Unexpectedly though, *FMR6* was silenced in both full and premutation mutation carriers, suggesting abnormal transcription and/or chromatin remodelling prior to transition to the full mutation (Khalil *et al.*, 2008). Both *FMR4* and *FMR6* are thought to regulate *FMR1* stability, splicing, subcellular localization and translational efficiency in FXTAS, and have been proposed to be useful biomarkers allowing for early detection and therapeutic intervention in fragile X syndrome and FXTAS. *FMR6*, in particular, may also be useful as a biomarker for FXPOI. In a study investigating the relationship between lncRNA accumulation and the pathophysiology of FXPOI, there was a non-linear association between the CGG-repeat number and *FMR6* expression in granulosa cells, with the highest levels of *FMR6* observed in women with mid-size CGG repeats (80–120) (Elizur *et al.*, 2016). In addition, the authors showed that increased *FMR6* expression had a negative relationship with the number of oocytes retrieved; no correlations were observed with *FMR4*. Thus the production of lncRNAs from the *FMR1* locus may be an additional pathway by which RNA can cause toxicity in FXPOI.

## Repeat-associated non-AUG translation: does it contribute to FXPOI?

Translation initiation is highly complex process requiring the step-wise assembly of elongation-competent 80S ribosomes at start codons of mRNA. In the canonical ribosome scanning model, successful translation of eukaryotic mRNA is thought to require components of the preinitiation complex to scan through the 5′UTR in the 5′ to 3′ direction, until it encounters an AUG codon in a good Kozak context, at which point, base-pairing between the AUG codon and the CAU anti-codon on tRNA^Met^ takes place. The 40S ribosome is now committed to its selection of start codon and is joined by the 60S ribosomal subunit, allowing the first peptide bond to be formed, thus beginning translation elongation (see Dever and Green, 2012 for a comprehensive review). In particular, dependency on the RNA helicase activity of eIF4A to resolve RNA secondary and tertiary structures is of significance, as these structures can impact translation initiation both positively and negatively depending on their location (Pestova and Kolupaeva, 2002). Furthermore, these structures can cause multiple atypical modes of initiation, one of which is the translation of mRNA initiated at non-AUG start codons.

RAN translation enables initiation and elongation in the absence of an AUG start codon, resulting in the production of multiple homopolymer-containing proteins, depending on the reading frame (Green *et al.*, 2016). Repeat sequences can drive RAN translation whether they are located within 5′UTRs, protein-coding reading frames, introns or non-coding RNAs. This non-canonical translational initiation process was discovered through the study of CAG trinucleotide expansions in the *ATXN8* gene, which causes the neurodegenerative disorder spinocerebellar ataxia type 8 (SCA8) (Zu *et al.*, 2011). Unexpectedly, mutation of the only AUG codon upstream of the coding sequence did not abolish translation, rather a series of homopolymeric proteins were generated with glutamine, serine or alanine repeats. This phenomenon was shown to be dependent on the stability of the RNA secondary structures formed as a result of the expanded CAG repeats, as decreasing the number of the repeats stopped RAN translation. Importantly, polyalanine-ATXN8 proteins have been observed in the cerebella of SCA8 human patients and mouse models, and a similar approach has provided evidence of a polyglutamine RAN product from the DMPK gene with expanded CAG repeats in myotonic dystrophy (Zu *et al.*, 2011). More recently it has been demonstrated that the CGG-repeats found in the 5′UTR of *FMR1* also support RAN translation initiation, thus this may be a potential protein-based mechanism that underlies the development of FXTAS and FXPOI (Todd *et al.*, 2013).

In FXTAS, RAN translation initiated within the 5′UTR can occur in at least two reading frames, yielding either a polyglycine product, named FMRpolyG, or a polyalanine product (FMRpolyA) (Todd *et al.*, 2013); however, it was demonstrated that translation predominantly occurs in the glycine frame through initiation at a near cognate ACG codon located upstream of the expanded CGG repeats (Fig. 2B) (Sellier *et al.*, 2017). In accordance with this, FMRpolyG protein deposits are found to accumulate in ubiquitin-positive inclusions in the brain tissue of humans with FXTAS (Todd *et al.*, 2013); it is unclear at present whether or not FMRpolyA is expressed *in vivo*. Furthermore, it should be noted that RAN translation can also occur on non-expanded repeats, thus it may have normal and pathogenic roles (Todd *et al.*, 2013). FMRpolyG expression is observed in the ‘Dutch’ premutation mouse, and turning off *FMR1* transgene expression in this model reverses the formation of neuronal FMRpolyG-positive inclusions and FXTAS behavioural deficits (Hukema *et al.*, 2015). It is also proposed that similar to toxic RNA aggregates, FMRpolyG can sequester specific proteins required for viable cell function through protein–protein interaction. Indeed, FMRpolyG has been shown to interact with LAP2B, a protein essential in anchoring lamina proteins to the inner nuclear membrane (Sellier *et al.*, 2017); overexpression of LAP2B rescues neuronal cell death induced by the expression of FMRpolyG. Furthermore, experiments in *Drosophila* and premutation cell line models suggest that FMRpolyG interferes with the ubiquitin proteasome system, and prevention of RAN translation can attenuate this impairment (Oh *et al.*, 2015). With regards to FXPOI, ubiquitin-positive inclusions were frequently observed at a high density within ovarian stromal cell nuclei from five women with the condition compared to stromal cells of four control ovaries, where these ubiquitin-positive inclusions were much rarer (Chang *et al.*, 2011). Furthermore, double labelling of ubiquitin-positive inclusions found in the stromal cells of one FXPOI patient revealed that the vast majority (>90%) of the inclusions also stained positive for FMRpolyG (Buijsen *et al.*, 2016). As the ovarian tissue of this woman with FXPOI did not contain any follicles, analyses of the ovaries of the ‘Dutch’ mouse model were undertaken; while the ovaries of 20-week-old ‘Dutch’ mice contained a few ubiquitin-FMRpolyG-positive inclusions, in 40-week-old ‘Dutch’ mice, similar to the FXPOI ovarian tissue, numerous intranuclear inclusions were detected in the ovarian stromal cells, both with ubiquitin and FMRpolyG antibodies (Buijsen *et al.*, 2016). Interestingly, in these studies, oocytes, granulosa and theca cells of follicles of all stages were negative for these inclusions, which is surprising as these are thought to be the primary cell types affected in FXPOI. It may be the case that loss of affected follicles may occur too quickly for the pathological inclusions to be visualized, analogous to the situation observed in Purkinje cells of FXTAS patients (Greco *et al.*, 2002). In a more recent study, however, FMRpolyG aggregates have been identified in mural granulosa cells from six *FMR1* premutation carriers, and these aggregates showed varying levels of co-localization with ubiquitin (Friedman-Gohas *et al.*, 2020). None of the granulosa cells from the four control women expressed FMRpolyG. Additionally, *in vitro* work transfecting plasmids expressing premutation-range CGG repeats into COV434 cells resulted in the formation of FMRpolyG aggregates (Friedman-Gohas *et al.*, 2020). Therefore, while this may provide some evidence that RAN translation and FMRpolyG expression might be linked to this premutation disease, significantly more data are necessary to establish conclusively whether FMRpolyG-inclusions are a pathological characteristic of FXPOI, and of mechanistic importance.

## Concluding remarks

The molecular mechanism underlying FXPOI is enigmatic, and while the study of FXTAS and other expanded-repeat disorders has provided some insight, the discovery of RAN translation adds an additional complexity as to whether CGG-repeats in the 5′UTR of *FMR1* elicit toxicity via RNA gain-of-function or protein-based means, or a combination of both. Transgenic mice expressing both CGG repeat RNA and FMRpolyG protein, but not mice expressing only the mutant RNA containing expanded CGG repeats, exhibit inclusion formation and motor phenotypes reminiscent of FXTAS (Sellier *et al.*, 2017), implying that RAN protein products play a role in the disease phenotype. However, the ‘130R’ mouse, which is unable to make polyglycine and polyalanine proteins due to the position of a stop codon in the upstream sequence, still has evidence of ovarian dysfunction, suggesting that at least some pathology must arise independently of RAN translation and could be related to the elevated levels of premutation *FMR1* mRNA. Furthermore, although data from the premutation mouse models show there is no detrimental effect on the ovarian reserve at birth, it is still a possibility that gonadotoxic RNA or protein aggregates may form during this crucial period, with the effects only becoming apparent in post-natal life. However, establishing the presence of RNA or protein aggregates in follicle structures may be difficult, given the limited access to such clinical specimens and the rapid loss of affected follicles in POI: only very limited ovarian histological evaluation has been reported in women with premutations (Chang *et al.*, 2011). Typical intranuclear inclusion formation has also been observed in neurons of the anterior pituitary with degenerative changes in gonadotropic cells (Greco *et al.*, 2007), raising the possibility of abnormal signalling via the hypothalamic–pituitary–gonadal axis. However, women with FXPOI have the characteristic elevated levels of FSH and LH (Murray *et al.*, 1999) as in other causes of POI, so these inclusions may not have any immediate relevance. Finally, it has been shown that *FMR1* mRNA in human and mouse brain is expressed as a combination of multiple isoforms that use alternative transcriptional start sites and different polyadenylation sites, and specific regulation of the UTRs is observed in the brains of premutation carriers (Tassone *et al.*, 2011; Tseng *et al.*, 2017). This suggests that transcript variants may play a role in pre-mutation related pathologies. Therefore, understanding the expression of these different isoforms will be fundamental as imbalances in their expression could underlie disease progression. Thus, while using FXTAS as a model to study FXPOI pathogenesis has had some benefit, the central questions which remain unanswered are the relative contributions of RNA gain-of-function and protein-based pathology to the overall disease phenotype, and the timing and location (at the cellular and sub-cellular level) of this insult in the ovary which results in follicle atresia and POI. Development of specific cell line and animal models to explore these questions will be key in illuminating the disease biology of FXPOI.

## Authors’ roles

R.R. wrote the manuscript; R.A.A. edited the manuscript; both authors approved the final version.

## Funding

The authors’ work in this field is support by grants from the Medical Research Council (G1100357 to R.A.A., MR/N022556/1 to the MRC Centre for Reproductive Health) and Wellbeing of Women (PRF005 to R.R.). 

## Conflict of interest

R.R. declares no conflict of interest. R.A.A. reports grants and personal fees from Roche Diagnostics and personal fees from Ferring Pharmaceuticals, IBSA, Merck Serono, outside the submitted work.
